# Abnormal Angle-Dependent Multi-Channel Filtering in Photonic Crystals Containing Hyperbolic Metamaterials

**DOI:** 10.3390/nano15141122

**Published:** 2025-07-19

**Authors:** Mingyan Xie, Yuanda Huang, Haoyuan Qin, Guiqiang Du

**Affiliations:** 1 SDU-ANU Joint Science College, Shandong University, Weihai 264209, China; xiemingyan@mail.sdu.edu.cn (M.X.); yuandahuang@mail.sdu.edu.cn (Y.H.); 2 School of Space Science and Technology, Shandong University, Weihai 264209, China; 202100830071@mail.sdu.edu.cn

**Keywords:** photonic crystal, hyperbolic metamaterial, optical filters, angle-independent

## Abstract

Tunneling modes in all-dielectric one-dimensional photonic crystals can be utilized for multi-channel filtering. However, these tunneling modes generally blue shift upon increasing the incident angle. When hyperbolic metamaterials are introduced into one-dimensional photonic crystals, the competition between the propagation phase shifts in the dielectric materials and hyperbolic metamaterials can result in different angle dependencies, including blue shift, abnormal zero shift, and abnormal red shift. When the reduction in the propagation phase in the dielectric layer exceeds the increment in the propagation phase in the hyperbolic metamaterial, the tunneling modes are blue-shifted; conversely, when the phase increment in the hyperbolic metamaterial exceeds the phase reduction in the dielectric layer, the tunneling modes are abnormally red-shifted. When the phase changes in the two materials are the same, the tunneling modes are angle independent. In this study, we investigated the multiple filtering effects of one-dimensional photonic structures composed of hyperbolic metamaterials. These composed structures exhibited multiple tunneling modes based on one-, two-, or three-angle dependencies and can be applied in novel optical devices with different angle-dependence requirements.

## 1. Introduction

Optical filters, including multi-channel filters [1,2,3,4], have attracted tremendous attention as important optical devices owing to their applicability in display devices, organic solar cells, refractive index sensors, and integrated fluorescence sensors [5,6,7,8]. As proposed in 1987, photonic crystals (PCs) are periodic nanostructures generally composed of different materials with varying refractive indices [9,10]. Owing to their photonic bandgap (PBG) and photonic localization [11,12], PCs have been applied in optical diodes, lasers, fibers, reflectors, absorbers, sensors, and structural color devices [13,14,15,16,17,18,19].

Leveraging the excellent optical properties of PCs, one-dimensional PCs (1D-PCs), which are the simplest nanostructures, can be used to design different types of optical filters, including narrowband optical filters [20] and omnidirectional optical filters [21], by adjusting the inner structures and material parameters. In recent years, researchers have designed different multi-channel filters based on 1D-PCs. In 2003, Qiao et al. designed a multi-channel filter based on the quantum well (QW) structure of 1D-PCs [2], and the number of tunneling modes in the spectra could be regulated by adjusting the period number inside the 1D-PCs. In 2021, Zhang et al. discovered the multi-channel filtering effect of 1D-PCs containing single-negative materials [3]. In the same year, Baseri et al. designed a filter based on 1D nano-superconductor PCs [4], and Tan et al. designed a highly sensitive solar-blind ultraviolet band-selective metal–semiconductor–metal photodetector based on PC filters [1]. However, the optical transmission properties of all-dielectric 1D-PCs are angle-dependent and blue-shifted owing to the multiple Bragg interference effects and the reduced propagation phase in the dielectric layer [16,22,23]. These types of angle-dependent optical filters can be applied in detectors, sensors, cloaks, and other devices widely used in various fields [18,24,25,26]. However, all-dielectric 1D-PCs cannot achieve angle-independent or red-shifted optical filtering, which limits their applications. In recent years, optical metamaterials have attracted considerable attention from researchers, and combining optical metamaterials with PCs is a promising approach for designing new optical devices [20,27]. Through reviewing previous studies on 1D-PC filters [20,24,28], it was found that some studies investigated the angle dependence of single-channel filtering effects, which restricted their potential applications compared to multi-channel filtering effects. However, existing studies on multi-channel filtering effects did not explore different angle dependences.

Optical metamaterials are artificial periodic optical nanostructures composed of subwavelength unit cells [29,30,31]. In the case of hyperbolic metamaterials (HMMs) [32,33,34], the sign of one principal component of the permittivity or permeability tensor is opposite to those of the other two components [30]. Unlike isotropic dielectric materials that exhibit circular iso-frequency curves, HMMs show hyperbolic iso-frequency curves [34]. Recent studies have revealed the notable properties and applications of HMMs, such as a negative refractive index, surface plasmon polariton modulation, and super-resolution imaging [35,36,37,38,39]. In addition, the properties of HMM-containing 1D-PCs, such as an abnormal zero-shift (angle-independent) PBG [30,40,41], an abnormal red-shift PBG [42,43,44], and wide-angle polarization beam-splitting effects, differ from those of all-dielectric 1D-PCs; these unique properties can be utilized to design various new optical devices.

In this study, we constructed 1D-PCs containing HMMs and achieved multiple kinds of multi-channel optical filtering effects with different angle dependencies. Based on the phase compensation theory [30], we analyzed the angle dependencies of the tunneling modes in the 1D-PC spectra containing HMMs. It was found that these tunneling modes had three different angle dependencies, and the tunneling modes with different angle dependencies could simultaneously appear in the spectra of 1D-PCs containing HMMs. The optical filtering properties of these 1D-PCs were different from those of all-dielectric 1D-PCs. These nanostructures have an important scientific significance for the development of new multi-channel optical filters in the future.

## 2. HMM-Containing 1D-PC-Based Optical Filters

**Figure 1:**
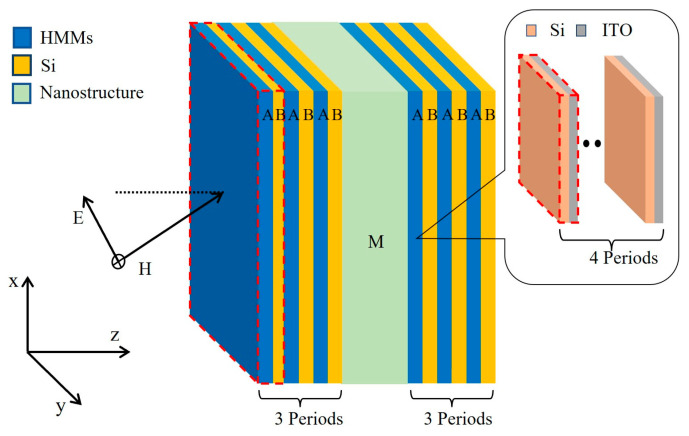
Schematic of the 1D-PC structure (AB)_3_M(AB)_3_, and inner periodic optical nanostructures of the HMM A is mimicked by a subwavelength Si/ITO multilayer (CD)_4_.

We considered 1D-PCs containing HMMs to realize different types of multi-channel optical filtering, denoted as (AB)_3_M(AB)_3_, where A, B, and M represent the HMM, dielectric material, and multilayer, respectively. Medium B is an isotropic dielectric, chosen as silicon, with a refractive index of *n*_Si_ = 3.48 [45]. The HMM A is composed of subwavelength metal–dielectric periodic multilayers, denoted as (CD)s, where C, D, and S represent the isotropic dielectric medium, metal, and period number, respectively. Here the medium C and D are selected to be Si and indium–tin oxide (ITO) [46]. The permittivity of ITO is similar to that of metals [47], which is described by the Drude model [48]:

(1) $${\varepsilon_{D}\left(\omega \right)=\varepsilon }_{\infty }-\frac{\omega_{pD}^{2}}{\omega^{2}+i\omega \gamma_{D}},$$

where $\varepsilon_{\infty }$ is the high-frequency permittivity with a value of 3.9 for ITO; $\omega_{pD}$ denotes the plasma frequency (here, we set $\hbar \omega_{pD}=2.48\;eV$ [30]); $\;\gamma_{D}$ is the damping frequency, and $\gamma_{D}$ is equal to zero in the lossless case, and when the loss of ITO is considered, the value of $\gamma_{D}$ satisfies the equation $\hbar \gamma_{D}=0.016\;eV$; and ω represents the angular frequency of the electromagnetic wave; The multilayer M is composed of an Si layer and HMM layer. Therefore, the optical properties of ITO materials are similar to those of metals in the near-infrared region [46]. Figure 1 presents a schematic of the structure of the 1D-PC (AB)_3_M(AB)_3_, where A is mimicked by the subwavelength periodic Si/ITO multilayer (CD)_4_; here, the incident angle *θ* is in the *x–z* plane, and A, B, C, and D denote the same layers in the following sections, respectively. These kinds of 1D-PCs containing HMMs can be fabricated layer-by-layer by ion-assisted electron beam evaporation under high vacuum, and similar experimental setups have been extensively studied [34,49,50]. The manufacturing process is as follows: Firstly, a high vacuum environment is formed, and the target material is melted and evaporated by electron beam heating; secondly, the evaporated material deposits on the surface of the substrate to form the thin film. By alternating the Si and ITO target materials and repeating the process, 1D-PCs containing HMMs can be constructed.

**Figure 2:**
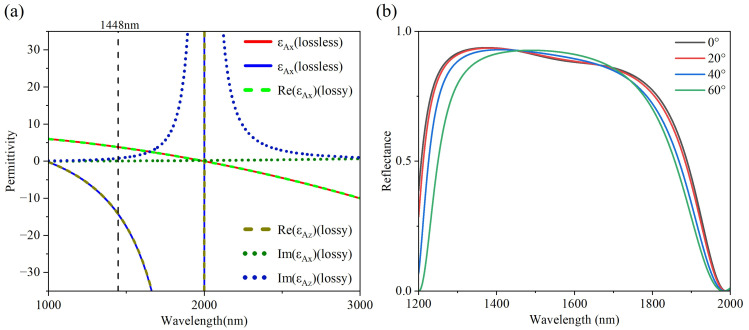
(**a**) Effective permittivity tensor of the HMM (CD)_4_ as a function of wavelength where the corresponding thicknesses of layer A and B are 200 nm and 98 nm at the Bragg wavelength. (**b**) Reflectance spectra of 1D-PCs containing HMMs (AB)_3_.

We utilized the Effective Medium Theory (EMT) to analyze the optical properties of the HMMs. The components of the effective permittivity tensor of (CD)_4_ are given by [51,52]:

(2) $$\varepsilon_{Ax}=\varepsilon_{C}\left(1-f\right)+\varepsilon_{D}f,$$

(3) $$\varepsilon_{Az}=\frac{1}{\frac{1-f}{\varepsilon_{C}}+\frac{f}{\varepsilon_{D}}},$$

where $f=d_{D}/(d_{C}+d_{D})=0.5$ represents the dielectric-layer filling ratio. We obtained the effective permittivity tensor of the subwavelength structure (CD)s as a function of wavelength, which is shown in Figure 2a. The shaded area in Figure 2 represents the type-I hyperbolic region, which is located at 1000–2000 nm. Here, two solid lines of different colors (red and blue) represent the variations of $\varepsilon_{Ax}$ and $\varepsilon_{Az}$ with wavelength, without considering the loss of the ITO material. Two dashed lines of different colors (green and olive) correspond to the real parts of $\varepsilon_{Ax}$ and $\varepsilon_{Az}$ when the loss of the ITO material is taken into account. Meanwhile, two dotted lines of different colors (dark green and blue) represent the imaginary parts of $\varepsilon_{Ax}$ and $\varepsilon_{Az}$ under the same loss-included condition. According to the phase compensation theory [30], Xue et al. calculated the thickness of HMMs and the dielectric for phase compensation:

(4) $$d_{A}=\frac{\pi c}{\omega_{Bragg}}\frac{1}{\sqrt{\varepsilon_{Ax}}(1-\frac{\varepsilon_{B}}{\varepsilon_{Az}})},$$

(5) $$d_{B}=\frac{\pi c}{\omega_{Bragg}}\frac{1}{\sqrt{\varepsilon_{B}}(1-\frac{\varepsilon_{Az}}{\varepsilon_{B}})},$$

The thickness of layer A and B is determined by the Bragg condition. At the Bragg wavelength $\lambda_{Bragg}$ in the first PBG, the Bragg condition is given by:

(6) $$\phi ={\left(k_{Az}d_{A}+k_{Bz}d_{B}\right)}_{\lambda_{Bragg}}=\pi ,$$

The term ϕ is the propagation phase in a unit cell of 1D-PCs (AB)_n_. The Bragg wavelength $\lambda_{Bragg}$ is selected to be 1448 nm [30]. Based on Equations (4) and (5), the thicknesses of the layers are calculated to be *d*_A_ = 200 nm and *d*_B_ = 96 nm, which is shown in Figure 2a, the periodic number S is calculated to be 4, and the thickness of C and D are $d_{C}=fd_{A}/4=25\;nm$ and $d_{D}=(1-f{)d}_{A}/4=25\;nm$. Consequently, the photonic structure (AB)_3_ containing HMMs can achieve angle-independent PBG, as illustrated in Figure 2b.

The transform matrix method [53] was used to simulate the optical properties of the 1D-PCs, and optical waves with transverse magnetic (TM) polarization were transmitted through the 1D-PCs. Generally, the shifting mechanism of the tunneling modes of the 1D-PCs is determined by [30]:

(7) $$\frac{\partial \phi }{\partial k_{x}}={\left(d_{A}\frac{\partial k_{Az}}{\partial k_{x}}+d_{B}\frac{\partial k_{Bz}}{\partial k_{x}}\right)}_{\lambda_{mode}},$$

where *k*_Az_ and *k*_Bz_ are the *z*-components of the wave vectors in layers A and B, respectively; *k*_x_ is the *x*-component of the wave vector; and *λ*_mode_ denotes the wavelength of the tunneling mode. The red-shifted tunneling modes are expressed as [42]:

(8) $$\frac{\partial \phi }{\partial k_{x}}={\left(d_{A}\frac{\partial k_{Az}}{\partial k_{x}}+d_{B}\frac{\partial k_{Bz}}{\partial k_{x}}\right)}_{\lambda_{mode}}>0,$$

The angle-independent tunneling modes are expressed as [40]:

(9) $$\frac{\partial \phi }{\partial k_{x}}={\left(d_{A}\frac{\partial k_{Az}}{\partial k_{x}}+d_{B}\frac{\partial k_{Bz}}{\partial k_{x}}\right)}_{\lambda_{mode}}=0,$$

In addition, the blue-shifted tunneling modes are expressed as:

(10) $$\frac{\partial \phi }{\partial k_{x}}={\left(d_{A}\frac{\partial k_{Az}}{\partial k_{x}}+d_{B}\frac{\partial k_{Bz}}{\partial k_{x}}\right)}_{\lambda_{mode}}<0,$$

**Figure 3:**
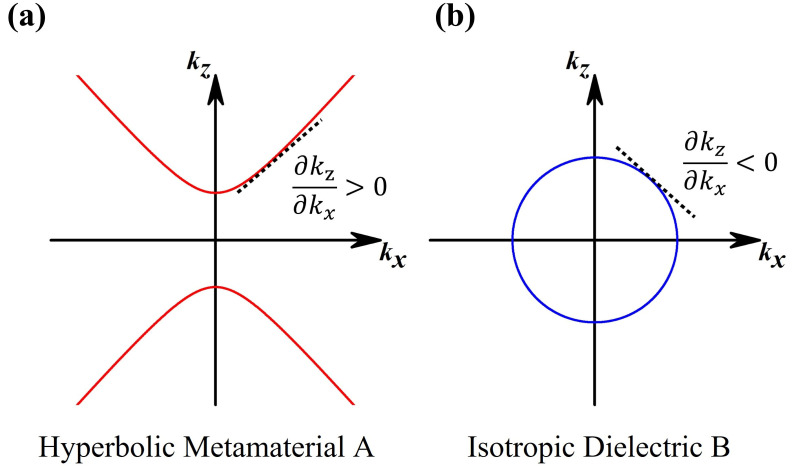
Iso-frequency curves of HMM A (**a**) and isotropic dielectric B (**b**) under TM polarization.

Herein, we consider 1D-PCs composed of HMMs (A) and dielectric materials (B). The shifting mechanisms of the tunneling modes, reflected in the optical spectra of these types of 1D-PCs, are related to the sign of $\frac{\partial \phi }{\partial k_{x}}$, which is determined by $\frac{\partial k_{Az}}{\partial k_{x}}$ and $\frac{\partial k_{Bz}}{\partial k_{x}}$. Figure 3 shows the iso-frequency curves of A (corresponding to the red line) and B (corresponding to the blue line). The equations for the TM-polarized iso-frequency curves of A and B are [42]:

(11) $$\frac{k_{x}^{2}}{\varepsilon_{Az}}+\frac{k_{Az}^{2}}{\varepsilon_{Ax}}=k_{0}^{2},$$

(12) $$k_{x}^{2}+k_{Bz}^{2}=\varepsilon_{B}k_{0}^{2},$$

which represent a hyperbola and circle, respectively; $\partial k_{Bx}/\partial k_{x}$ is negative, whereas $\partial k_{Az}/\partial k_{x}\;$ is positive, in the *k_x_* > 0 and *k_z_* > 0 domains. The opposite signs of the two terms in the same domain result in competition in the propagation phase. When the reduction in the propagation phase of B is greater than the increment in the propagation phase of A, the tunneling modes are blue shifted; by contrast, when the phase increment in A is greater than the phase reduction in B, the tunneling modes are abnormally red shifted. However, when the phase changes in the two materials are the same, the tunneling modes are angle independent.

In this study, we systematically investigated the transmission filtering characteristics of the 1D-PC (AB)_3_M(AB)_3_. By adjusting the multilayer M, the tunneling modes in the different 1D-PCs showed various angle dependencies depending on the different physical mechanisms where multiple tunneling modes revealed one-, two-, or three-angle dependencies. The classification of the results is also based on the types of angle dependences exhibited by the tunneling modes in the optical spectra. These nanostructures are crucial for developing new multi-channel optical filters.

## 3. Results

### 3.1. Multi-Channel Filtering Based on One Type of Angle-Dependence

By changing the multilayer M, we first investigated the multi-channel filtering effects with one type of angle dependence, including multi-channel blue shift, multi-channel red shift, and multi-channel angle-independent filtering effects. The structures of multilayer M are designed as (BABB)_3_, (AAP)_2_, and (QAP), where P and Q are both Si with thicknesses of *d*_P_ = 48.0 nm and *d*_Q_ = 115.2 nm. The corresponding 1D-PC structures are (AB)_3_(BABB)_3_(AB)_3_, (AB)_3_(AAP)_2_(AB)_3_, and (AB)_3_(QAP)(AB)_3_, respectively.

**Figure 4:**
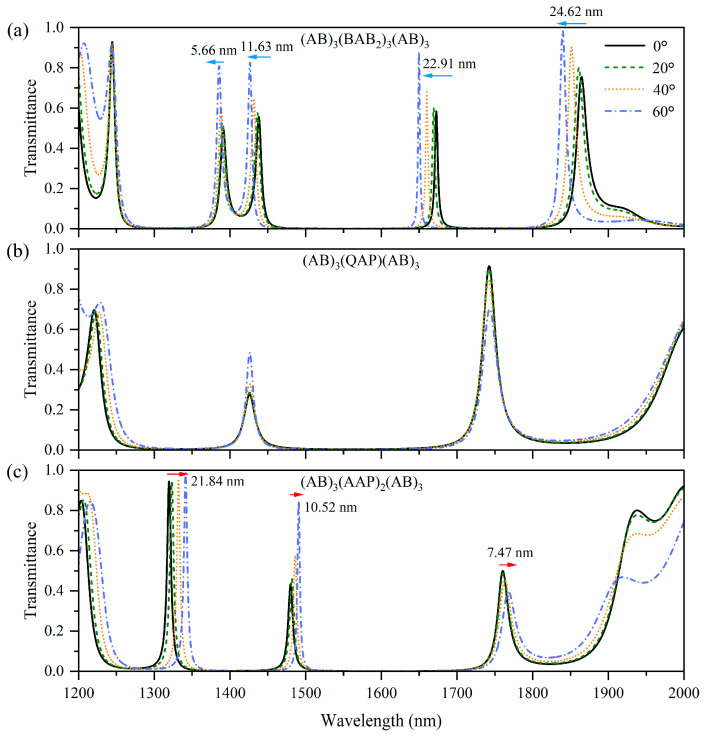
Simulated transmission spectra of the 1D-PCs (AB)_3_(BABB)_3_(AB)_3_ (**a**), (AB)_3_(AAP)_2_(AB)_3_ (**b**), and (AB)_3_(QAP)(AB)_3_ (**c**) at incident angles 0°, 20°, 40°, and 60° under TM polarization.

Figure 4a–c shows the transmission spectra of TM waves for these 1D-PCs at incident angles of 0°, 20°, 40°, and 60°. The transmission spectrum of (AB)_3_(BABB)_3_(AB)_3_ (Figure 4a) shows three tunneling modes that exhibit blue-shifted characteristics, and the wavelengths of these tunneling modes decrease by 5.66 nm, 11.93 nm, 22.91 nm, and 24.62 nm, respectively, with increasing incident angle. Unlike all-dielectric 1D-PCs multi-channel filters, the degree of the blue shift in these 1D-PCs containing HMMs can be adjusted by modifying the multilayer M. Although the introduction of HMMs increases the propagation phase, the overall propagation phase of the HMM-containing nanostructures still decreases as the incident angle increases. Because the tunneling modes at different wavelengths satisfy Equation (10), all the tunneling modes remain blue shifted. For (AB)_3_(QAP)(AB)_3_ (Figure 4b), the amount by which the propagation phase increases is larger than that shown in Figure 4a, enabling phase compensation. Consequently, the tunneling modes at the two wavelengths satisfy Equation (9) such that two angle-independent peaks appear in the transmission spectrum. In the case of (AB)_3_(AAP)_2_(AB)_3_ (Figure 4c), the amount by which the propagation phase increases exceeds those shown in Figure 4a,b; therefore, the total propagation phase of the nanostructure increases, and Equation (8) is satisfied, where all of the tunneling modes red shift with the increasing incident angles, and the shift amounts are about 21.84 nm, 10.52 nm, and 7.47 nm toward the long wavelengths, respectively. Therefore, for 1D-PC multi-channel filters containing HMMs, the multilayer M can be adjusted to modify its contribution to the propagation phase, thereby reducing the degree of the blue shift and achieving phase compensation, or even realizing and enhancing the abnormal red shift.

### 3.2. Multi-Channel Filtering Based on Two Different Angle-Dependencies

The shifting mechanisms in the optical spectra of 1D-PCs are governed by Equation (7). This equation indicates that the tunneling modes at different wavelengths can exhibit different angular dependencies. Multiple tunneling modes in (AB)_3_M(AB)_3_ exhibit two different angle-dependencies when certain structures of the multilayer M are selected.

**Figure 5:**
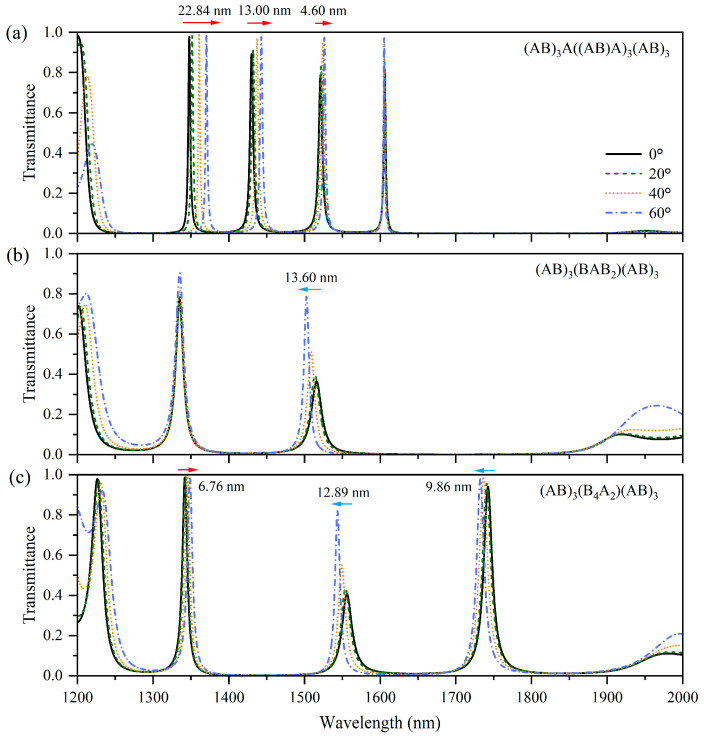
Simulated transmission spectra of the 1D-PCs (AB)_3_A((AB)A)_3_(AB)_3_ (**a**), (AB)_3_(BAB_2_)(AB)_3_ (**b**), and (AB)_3_(B_4_A_2_)(AB)_3_ (**c**) at incident angles 0°, 20°, 40°, and 60° under TM polarization.

Similarly, the multilayers M are designed as A((AB)A)_3_, (BAB_2_), and (B_4_A_2_), and the corresponding 1D-PCs are denoted by (AB)_3_A((AB)A)_3_(AB)_3_, (AB)_3_(BAB_2_)(AB)_3_, and (AB)_3_(B_4_A_2_)(AB)_3_, respectively. Their TM-wave transmission spectra at incident angles of 0°, 20°, 40°, and 60° are shown in Figure 5a–c. The transmission spectra of (AB)_3_A((AB)A)_3_(AB)_3_ (Figure 5a) show four tunneling modes. The remaining left three tunneling modes are red shifted; the shift amounts are about 22.84 nm, 13.00 nm, and 4.60 nm toward the long wavelengths, respectively, implying that these three tunneling modes satisfy Equation (8) at their corresponding wavelengths. The rightmost tunneling mode exhibits an angle-independent behavior, implying that this tunneling mode satisfies Equation (9). The degree of the red shift for these three tunneling modes gradually decreases as the wavelengths increase, indicating that a longer incident wavelength leads to a reduction in the propagation phase shift variation, and the angle dependence of the tunneling mode evolves from Equation (8) to Equation (9); thus, two different angle dependencies can be obtained in one structure. In the transmission spectra of (AB)_3_(BAB_2_)(AB)_3_ (Figure 5b), the leftmost tunneling mode is angle independent. The tunneling mode on the right shifts 13.60 nm toward shorter wavelengths. Similarly, an increase in the incident wavelength reduces the variations in the propagation phase shift, resulting in the angle dependence of the tunneling mode transferring from Equation (9) to Equation (10). In the transmission spectra of (AB)_3_(B_4_A_2_)(AB)_3_ (Figure 5c), the leftmost tunneling mode is red shifted; this tunneling mode shifts 6.76 nm toward the long wavelength, whereas the other two tunneling modes shift 12.89 nm and 9.86 nm toward short wavelengths. Owing to the reduced propagation phase shift, the angular dependence of the tunneling mode evolves from Equation (8) to Equation (10). Therefore, a multi-channel filter based on two angle dependencies can be realized, and the degree of the angle dependence of each tunneling mode can be controlled. These types of 1D-PCs have important potential applications in optical filters with different angle dependencies at different operating wavelengths.

### 3.3. Multi-Channel Filtering Based on Three Different Angle-Dependences

**Figure 6:**
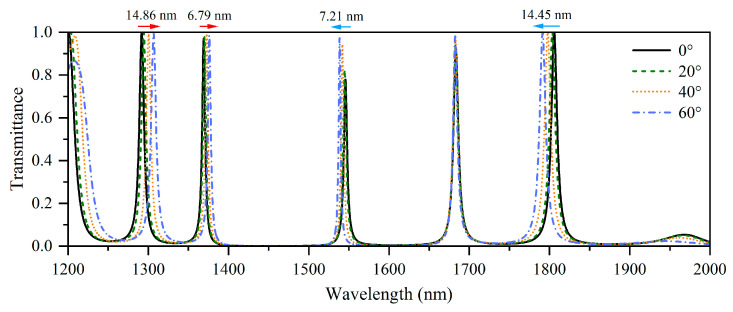
Simulated transmission spectrum of the 1D-PC (AB)_3_(A_3_B_5_A_2_)(AB)_3_ at the incident angles 0°, 20°, 40°, and 60° under TM polarization.

The multilayer M is chosen as A_3_B_5_A_2_, and the corresponding 1D-PC is denoted by (AB)_3_(A_3_B_5_A_2_)(AB)_3_. Figure 6 shows the TM-wave transmission spectra of the 1D-PC at incident angles of 0°, 20°, 40°, and 60°. The tunneling modes in this nanostructure exhibit a rich variety of angle dependencies, where red-shifted, blue-shifted, and angle-independent modes appear simultaneously.

Five tunneling modes are observed in Figure 6, where the shift amounts of two left tunneling modes approximately 14.86 nm and 6.79 nm toward the long wavelengths as the angle increases because Equation (9) is satisfied at their corresponding wavelengths. The second tunneling mode on the left exhibits a smaller red shift than does the first, indicating that its propagation phase is smaller than that of the first tunneling mode. The third tunneling mode on the left moves 7.21 nm toward shorter wavelengths as the angle increases, implying that this mode is blue shifted because Equation (10) is satisfied. However, the fourth tunneling mode on the left is angle independent, implying that the propagation phase increases from the third to the fourth tunneling mode. Otherwise, the rightmost tunneling mode shows a blue shift; this tunneling mode shifts about 14.45 nm toward the short wavelength which implies that Equation (8) is non-monotonic. Therefore, by adjusting the multilayer M, multiple tunneling modes in HMM-containing 1D-PCs can present different angle dependencies, and the degree of these angle dependencies can be precisely controlled. These nanostructures are important for fabricating new optical filters with multiple angle dependencies.

## 4. The Influence of Loss on 1D-PCs Filtering

In Section 3, we investigated the angle dependence of filtering effects in 1D-PCs containing HMMs under lossless conditions ( $\gamma_{D}$ = 0). These tunneling modes, which can be utilized for filtering, exhibit a more pronounced and complicated angle dependence compared to the PBGs in the 1D-PCs. However, the $\gamma_{D}$ of ITO materials is a non-zero value. Therefore, it is necessary to evaluate the influence of loss characteristics of ITO on the previously discussed filtering effects.

**Figure 7:**
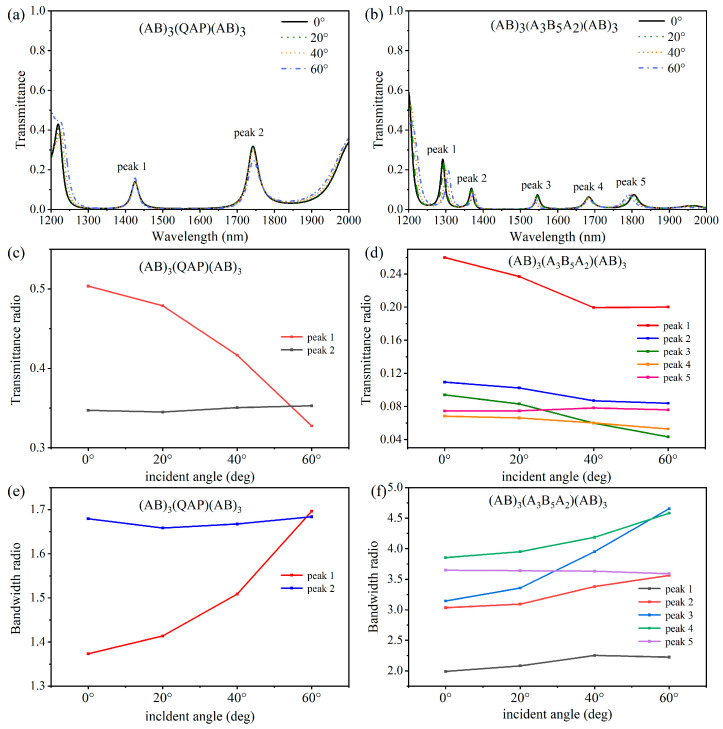
(**a**) Simulated transmission spectra of the 1D-PC (AB)_3_(QAP)(AB)_3_ at the incident angles 0°, 20°, 40°, and 60° under TM polarization. (**b**) Simulated transmission spectra of the 1D-PC (AB)_3_(A_3_B_5_A_2_)(AB)_3_ at the incident angles 0°, 20°, 40°, and 60° under TM polarization. (**c**) The transmittance ratios (loss/lossless) at the peak wavelength of two tunneling modes of 1D-PC (AB)_3_(QAP)(AB)_3_ at the incident angles 0°, 20°, 40°, and 60°. (**d**) The transmittance ratios at peak wavelength of five tunneling modes of 1D-PC (AB)_3_(A_3_B_5_A_2_)(AB)_3_ at the incident angles 0°, 20°, 40°, and 60°. (**e**) The bandwidth ratios of two tunneling modes of 1D-PC (AB)_3_(QAP)(AB)_3_ at the incident angles 0°, 20°, 40°, and 60°. (**f**) The bandwidth ratios of five tunneling modes of 1D-PC (AB)_3_(A_3_B_5_A_2_)(AB)_3_ at the incident angles 0°, 20°, 40°, and 60°.

In this section, the value of $\gamma_{D}$ is considered as 0.016 eV/ℏ. Normally, when $\gamma_{D}$ takes a non-zero value, it affects not only the imaginary part of $\varepsilon_{D}$ but also changes the real part of $\varepsilon_{D}$. However, its impact on the real part of $\varepsilon_{D}$ is negligible. This is evident from Figure 2, where the curves depicting the real parts of $\varepsilon_{Az}$ and $\varepsilon_{Ax}$, considering the loss effect of ITO, nearly overlap with those under the lossless conditions. Therefore, although the loss in ITO affects the transmittance of 1D-PCs, their influence on the angle dependence of the tunneling modes within 1D-PCs is negligible. Here, Figure 7 gives the optical spectra of the 1D-PCs (AB)_3_(QAP)(AB)_3_ and (AB)_3_(A_3_B_5_A_2_)(AB)_3_.

Figure 7 demonstrates that the optical loss characteristics of ITO affect the transmission spectrum of the 1D-PCs. As shown in Figure 7a,b, the loss of ITO significantly affects the transmittance of the 1D-PC at these tunneling modes, which increases the difficulty of applying this type of photonic nanostructure in filtering. We calculated the transmittance ratios of 1D-PCs (AB)_3_(QAP)(AB)_3_ and (AB)_3_(A_3_B_5_A_2_)(AB)_3_ considering the loss compared to that without the loss at the central wavelengths of the tunneling modes under different incident angles, as shown in Figure 7c,d. The results show that the loss of ITO significantly reduces the transmittance of the tunneling mode, especially in 1D-PC (AB)_3_(A_3_B_5_A_2_)(AB)_3,_ as shown in Figure 7b.

We also evaluated the loss impact of ITO on the bandwidth so that we could calculate the bandwidth ratios of tunnel modes in 1D-PCs (AB)_3_(QAP)(AB)_3_ and (AB)_3_(A_3_B_5_A_2_)(AB)_3_ with the incident angle, as shown in Figure 7e,f. The results show that the loss increases the bandwidths of tunneling modes in 1D-PCs. However, the angle dependence of these tunneling modes remains nearly unaffected since the resonant wavelengths show negligible shifts. Therefore, we need to strike a balance between the requirements for the working performance of the filters and the requirements for the angle dependences of the filters.

## 5. Conclusions

In this study, we investigated the abnormal multiple optical filtering properties of 1D-PCs containing HMMs based on different physical mechanisms. Based on these properties, several types of 1D-PCs containing HMMs were designed. These photonic structures can realize 2–5 channel filtering effects, and these multi-channel optical filtering effects of the 1D-PCs displayed 7 kinds of angle dependencies: all the tunneling modes were blue shifted, red shifted, and angle independent; sectional tunneling modes were red shifted, whereas others were angle independent; sectional tunneling modes were blue shifted, whereas others were angle independent; sectional tunneling modes were blue shifted, whereas others were red shifted; sectional tunneling modes were blue shifted; and sectional tunneling modes were red shifted, whereas others were angle independent. In our results, the red-shifted tunneling modes exhibited a minimum shift of 4.60 nm and a maximum shift of 22.84 nm toward the longer wavelengths. Conversely, the blue-shifted tunneling modes demonstrated a minimum shift of 5.66 nm and a maximum shift of 24.62 nm toward shorter wavelengths. These proposed 1D-PCs are expected to aid in the development of new multi-channel optical filters. This work is expected to be promoted to 2D-PCs and 3D-PCs in the future.

## Data Availability

The data that support the findings of this study are available upon reasonable request from the authors.
